# The Role of RAS in CNS Tumors: A Key Player or an Overlooked Oncogene?

**DOI:** 10.3390/ijms26094104

**Published:** 2025-04-25

**Authors:** Isabel de Souza Barbosa, Carlos Pilotto Heming, Vivaldo Moura Neto, Veronica Aran

**Affiliations:** 1 Laboratório de Biomedicina do Cérebro, Instituto Estadual do Cérebro Paulo Niemeyer, Secretaria Estadual de Saúde, Rio de Janeiro 20261-901, Brazil; isabelsbarbosa13@gmail.com (I.d.S.B.); cp.heming@gmail.com (C.P.H.); vivaldomouraneto@gmail.com (V.M.N.); 2 Programa de Pós-Graduação em Anatomia Patológica, Faculdade de Medicina, Universidade Federal do Rio de Janeiro (UFRJ), Rio de Janeiro 21941-853, Brazil; 3 Laboratório de Morfogênese Celular (LMC), Instituto de Ciências Biomédicas (ICB), Universidade Federal do Rio de Janeiro (UFRJ), Rio de Janeiro 21941-853, Brazil

**Keywords:** RAS mutations, CNS tumors, glioblastoma, oncogenic signaling, targeted therapy

## Abstract

This review examines the prevalence, molecular mechanisms, and clinical implications of *RAS* mutations in Central Nervous System (CNS) tumors, with a particular focus on glioblastoma. We summarize the current understanding of RAS-driven oncogenic pathways, their contribution to tumor progression, and potential therapeutic strategies targeting *RAS* and its downstream effectors. Although direct *RAS* mutations are rare in primary CNS tumors, alterations in RAS signaling, such as *NF-1* loss and aberrant receptor tyrosine kinase activation, contribute to malignant progression. Furthermore, emerging evidence links *RAS* mutations to brain metastases, highlighting their significance in CNS oncology. We also discuss recent clinical trials investigating RAS-targeted therapies, including covalent inhibitors, MEK inhibitors, and novel combination approaches. Given the increasing recognition of RAS pathway alterations in CNS malignancies, further research is needed to elucidate their role in tumor biology and explore targeted therapeutic interventions.

## 1. Introduction

Malignant tumors of the central nervous system (CNS), arising from abnormal tissue growth in the brain and spinal cord, account for only 1.6% of all tumors, yet have high mortality rates, being responsible for 2.5% of tumor-related deaths, according to the World Health Organization (WHO) [1]. Malignant CNS tumorigenesis is frequently associated with oncogenic signaling, where different genes are involved, including genes belonging to the MAP kinase pathway [2].

In 1982, *HRAS* was designated as an oncogene for the first time due to a point mutation. Shortly thereafter, *NRAS* and *KRAS* were promptly discovered [3]. Since then, there has been significant dedication to the study of *RAS* [4]. Mutant *RAS* is one of the most frequent oncogenes in the context of human cancers, affecting approximately 19% of tumors [5].

RAS proteins have the ability to regulate cell proliferation, differentiation, and survival through their signaling networks [6]. When these pathways are deregulated, they contribute to a malignant tumor phenotype characterized by uncontrolled cell growth, invasion, resistance to programmed cell death and angiogenesis [7]. There are three main RAS isoforms, *KRAS*, *NRAS* and *HRAS*, which encode four isoforms (*HRAS*, *NRAS*, *KRAS4A* and *KRAS4B*), widely expressed in human cells, having 82–90% homology in their sequence [8]. Oncogenic mutations usually affect codons 12, 13 and 61 and their mutations are usually of the missense type found mainly in the exon 2 [9]. *KRAS* is the Kirsten rat sarcoma viral oncogene homolog, codified by 6 exons localized in chromosome 12p12.1; *HRAS* is the Harvey rat sarcoma viral oncogene homolog localized in chromosome 11p15.5; and *NRAS* is the neuroblastoma viral oncogene homolog on chromosome 1p13.2 [10,11]. They encode GTPases that transmit signals by binding to GTP molecules and are inactivated when GTP is converted to GDP [12]. Single amino acid substitutions are responsible for mutations that confer enhanced GTP binding and reduced GTP to GDP conversion increasing activation of RAS proteins, thus altering cell signaling, causing excessive proliferation and consequent tumor formation [13].

The majority of *RAS* gene mutations (approximately 75%) occur in the *KRAS* gene, followed by *NRAS* mutations, which represent about 17%. Lastly, *HRAS* mutations account for around 7% of all *RAS* gene mutations [14]. *KRAS* mutations are most prominently found in pancreatic, colon, intestinal and lung adenocarcinomas [15]. Mutations in *HRAS* and *NRAS* are frequently present in head and neck, skin and hematopoietic cancers [11].

Human *KRAS* gene produces two distinct alternative splicing variants in exon 4, *KRAS4A* and *KRAS4B*, which differ mainly in their C-terminal region [16]. Exons 4A and 4B encode 39 and 38 amino acids, respectively, with 19 identical and 4 conserved substitutions [10]. Although for a long time *KRAS4B* was considered the main isoform because it is ubiquitous expressed in cancer [17], the *KRAS4A* isoform was also shown to be widely expressed in tumor cell lines, at levels comparable to *KRAS4B* in colorectal tumors. In murine models, *KRAS4A* proved to be dispensable: deletion of exon 4A resulted in viable embryos, which contrasts with the mortality observed with *KRAS4B* deletion [18].

## 2. RAS Signaling

RAS protein is involved in multiple intracellular signaling pathways, which are generally categorized into two main types: canonical (or classical) and non-canonical. Prominent examples of canonical pathways include the RAF/MEK/ERK cascade, also referred to as the MAPK pathway, and the PI3K/AKT/mTOR signaling route. In contrast, non-canonical pathways encompass less conventional signaling mechanisms, such as Tiam-RAC1 and PLCε [19]. In the case of RAF/MEK/ERK signaling pathway, it is initiated when growth factors bind to membrane receptors like EGFR or FGFR, triggering intracellular events that activate RAS via GTP exchange, assisted by adaptor proteins such as GRB2 and SOS1. Activated RAS then recruits RAF kinases (ARAF, BRAF, CRAF) to the membrane, where they activate MEK1/2, which subsequently activate ERK1/2. ERKs influence key cellular functions: in the nucleus, they regulate transcription factors (e.g., ELK1, MYC, c-Fos) involved in cell proliferation and differentiation; in the cytoplasm, they modulate the cytoskeleton and cell cycle regulators to adapt to the environment [20]. Another important pathway, the PI3K/AKT/mTOR pathway, is essential for regulating cell growth, metabolism, and survival. Activated by external signals downstream of RAS, it promotes proliferation and inhibits apoptosis. Briefly, RAS-GTP activates PI3K, which converts PIP2 into PIP3, recruiting AKT and PDK1. Activated AKT then stimulates mTORC1, enhancing anabolic processes. This pathway interacts with others, such as RAS/RAF/MEK/ERK, AMPK (which inhibits mTORC1 under energy stress), and HIF-1α in hypoxia. Given its central role in cancer, it is a key therapeutic target [21,22].

Non-canonical RAS signaling refers to alternative pathways through which RAS influences cell behavior, particularly in adhesion, cytoskeletal dynamics, and metabolism. Notable examples include the Tiam1-RAC1 pathway, where RAS activates Tiam1, a GEF for RAC1, promoting cytoskeletal reorganization and cell migration, and the PLCε pathway, where RAS stimulates PLCε to generate DAG and IP3, enhancing calcium signaling and activating PKC, thereby affecting proliferation, secretion, and stress responses [23,24]. Furthermore, non-canonical RAS signaling plays a crucial role in scenarios where classical pathways, such as RAF/MEK/ERK or PI3K/AKT, are inhibited—particularly during targeted therapy. In these cases, cancer cells may exploit alternative RAS effectors to maintain growth and survival, contributing to therapeutic resistance and disease progression through signaling rewiring [6].

RAS signaling pathways engage in significant cross-talk with other signaling axes, particularly receptor tyrosine kinases (RTKs). Among these, the platelet-derived growth factor (PDGF) ligands and their corresponding receptors, PDGFRα and PDGFRβ, are critical for proper nervous system development during embryogenesis, as well as for maintaining neuronal integrity and function in adulthood [25]. Upon binding of a dimerized PDGF ligand, receptor dimerization is induced, which in turn leads to autophosphorylation of intracellular tyrosine kinase domains. This phosphorylation event initiates the activation of several downstream signaling cascades, including pathways mediated by PLCγ, PI3K/AKT, RAS, SRC, and PKC [26].

Interestingly, members of the RAS family preferentially activate downstream effectors differently. HRAS effectively activates PI3K, KRAS strongly activates RAF, while NRAS activates both pathways moderately [27]. Several studies have found that the wild-type allele of mutated *RAS* gene suppresses tumor formation. Additionally, many *RAS*-mutated cancers exhibit loss of heterozygosity (LOH) at the mutated gene, indicating that losing the WT allele provides a growth advantage. Model systems for all three *RAS* genes have shown LOH to be a common event in cancer initiation [28,29].

*RAS*-mutated cancers are resistant to standard chemotherapy [30] necessitating new strategies to reach therapy efficiency [31,32]. A deep understanding of mutant *RAS* signaling is crucial for developing indirect targeting and combination therapies to improve treatment for these patients [27]. This review aims to synthesize current knowledge on the prevalence, molecular mechanisms, and clinical implications of *RAS* mutations in CNS tumors, an area with limited research contributions. This is clear when a simple search on *PubMed* is made with keywords “RAS “and “brain tumors”, with only 215 results appearing (PubMed Access: 1 February 2025). Furthermore, the text highlights the role of *RAS* mutations in tumor development and progression, explores potential treatment options, and identifies gaps in existing CNS research.

## 3. RAS and the CNS

Brain tumors encompass over 120 distinct diseases with varying histological, demographic, clinical, and molecular characteristics, within which glioblastoma (GBM) is the most common and aggressive malignant brain tumor in adults, remaining incurable [33]. Based on WHO CNS5 (2021), the latest classification [1], adult-type diffuse gliomas are astrocytoma, *IDH*-mutant; oligodendroglioma, *IDH*-mutant and 1p/19q-codeleted; and GBM, *IDH*-wildtype. *IDH*-mutant diffuse astrocytomas are graded 2–4 within their type; in addition, in the case that an IDH-mutant diffuse astrocytoma exhibits CDKN2A/B homozygous deletion, it is classified as a CNS WHO grade 4 tumor [1]. The presence of any one of the following five features is sufficient to classify an *IDH*-wildtype diffuse astrocytic glioma as a GBM: microvascular proliferation, necrosis, *TERT* promoter mutation, *EGFR* gene amplification, or combined whole chromosome 7 gain and 10 loss (+7/−10). If an *IDH*-wildtype tumor lacks these histological or molecular characteristics—appearing lower grade than a CNS WHO grade 4 GBM—it should be diagnosed as diffuse [1]. Examples of standard treatment include surgical resection, radiotherapy, and temozolomide (TMZ) chemotherapy [34,35]. Among various other CNS tumors, meningiomas and brain metastases are notable, with the latter exhibiting *RAS* alterations more frequently than the former [1].

*KRAS* is ubiquitously expressed in the human brain [36]. Its physiological function is mainly related to synaptic signaling in neurons [37]. Nevertheless, they have already been described as playing an essential role in gliomagenesis in mice [38,39]. Even though low in prevalence, *RAS* mutations have been correlated with increased *RAS* activation with the proliferation of malignant gliomas [38] and progression from low to high grade glioma [39]. Dysfunctional tumor signaling can arise not only from gene mutations, but epigenetic changes or pathway rewiring, which likely explains the lack of dominant driver mutations in some tumors, such as GBM [35]. Furthermore, a brain tumor model in rats included the *RAS* oncogene, which appeared to be important for the initial stages of neoplastic transformation, requiring additional genetic alterations for tumor progression [40].

Despite the fact that *RAS* mutations do not play a defining role in the classification or development of particular CNS tumor types [13], they still exist. For example, *RAS* mutations were observed in two glioma subtypes; in grade 2 oligodendrogliomas, *KRAS* mutations occurred in 4% of cases, while in GBM, they were present in approximately 1% [41]. Makino and colleagues detected four *RAS* mutations in gliomas: three in *KRAS* and one in *NRAS*. These included the *KRAS*^E76D^ mutation in an anaplastic astrocytoma *IDH*-wildtype, *KRAS*^G12A^ in an *IDH*-mutant anaplastic oligodendroglioma with 1p/19q codeletion, *KRAS*^Q61K^ in a ganglioglioma, and *NRAS*^Q61R^ in an *IDH*-wildtype anaplastic astrocytoma [13]. Interestingly, mutations in *PIK3CA* and *KRAS* were shown to lead to increased aggressiveness in gliomas and tumor progression independent of *IDH* mutational status [42]. In addition, a comprehensive review of cancer databases highlighted the frequency of *RAS* mutations in gliomas. GBM exhibited *HRAS^Q61^*, *KRAS^G12^*, *KRAS^Q61^*, *KRAS^A146^*, *NRAS^G12^*, *NRAS^Q61^* mutations, with respective incidences of 0.5%, 0.8%, and 0.6%. In low-grade gliomas, mutations included *KRAS^G12^*, *KRAS^G13^*, *KRAS^Q61^*, *NRAS^G12^*, *NRAS^G13^* and *NRAS^Q61^*, with frequencies of 0.1%, 1.0%, and 0.6% [5].

Recent findings associate *KRAS* mutations with arteriovenous malformations of the brain. The *KRAS*^G12V^ mutation, specifically, was linked to increased ERK activity, angiogenesis, Notch signaling, and migration—hallmarks of cancer progression [43]. Gliomas bearing *RAS* mutations, although reported, remain understudied [44,45,46]. One study highlighted that individuals with *KRAS* rs7212175 G > A polymorphism genotype have higher susceptibility to developing gliomas [2]. Additionally, mutant *KRAS* has been implicated in radiation-associated brain tumorigenesis [47]. Notably, our group identified a *KRAS* mutation in a pituitary macroadenoma, marking its first documentation in such a tumor type [48]. Conversely, no oncogenic *RAS* mutations were found in medulloblastoma [49].

Although not found to be prevalent in primary CNS tumors, *KRAS* mutations have been increasingly associated with brain metastases. It has been established that patients with *KRAS* mutations have a greater chance of developing brain metastases [50]. The hotspot mutation *KRAS^G12D^* was identified in the cerebrospinal fluid (CSF) of an individual with brain metastases from colon adenocarcinoma, while *NRAS^Q61L^* was detected in the plasma of a patient with brain metastases originating from melanoma [51]. In cases of brain metastases related to melanoma, the overall survival of the patient was found to be reduced in tumors presenting the *KRAS* mutation [52]. In a case series involving two lung cancer patients, CSF analysis of *KRAS* mutations aided in the early detection of leptomeningeal metastasis [53]. *KRAS* mutations activate EGFR signaling pathways, which are associated with an increased risk of brain metastases in non-small cell lung cancer (NSCLC) patients [54]. Nonetheless, they seem to indicate worse overall survival in these patients [55]. This can be mitigated with the administration of immune checkpoint inhibitors [56]. A recent case report has described complete disease remission of a *KRAS*-mutated brain oligometastatic lung cancer patient after immuno-chemotherapy with pembrolizumab [57].

**Figure 1:**
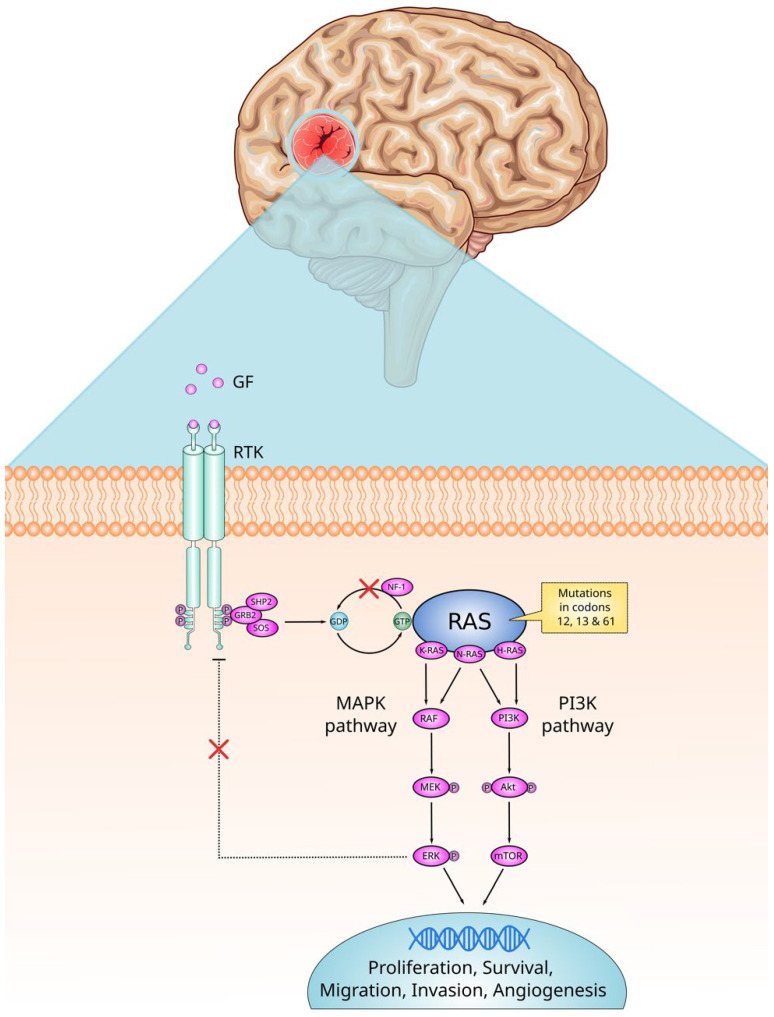
Oncogenic signaling pathways affected by mutant *RAS* and how they may relate to the development of CNS tumors. Upon binding to growth factors (GF), receptor tyrosine kinase (RTK) is activated. Guanine exchange factors act as mediators of the conversion of the RAS from an inactive GDP-bound state to an active GTP-bound state. Active RAS stimulate cell proliferation, survival, migration, invasion and angiogenesis by inducing signal transduction mainly via the MAPK and PI3K pathways. The mutation keeps the RAS in its active state and prevents NF-1 from hydrolyzing GTP. As a result, the signaling pathways are permanently activated, thus promoting tumorigenesis. Elevated levels of ERK negatively regulate RTK activation. However, constitutive activation of the RAS indicates negative feedback regulation fails. Akt = protein kinase B; ERK = extracellular signal-regulated kinase; GDP = guanosine diphosphate; GF = growth factor; GRB2 = growth factor receptor-bound protein 2; GTP = guanosine triphosphate; HRAS = Harvey rat sarcoma viral oncogene homolog; KRAS = Kirsten rat sarcoma viral oncogene homolog; MAPK = mitogen-activated protein kinase; MEK = mitogen-activated protein kinase; mTOR = mammalian target of rapamycin; NF-1 = neurofibromin 1; NRAS = Neuroblastoma rat sarcoma viral oncogene homolog; P = phosphate group; PI3K = phosphoinositide 3-kinase; RAF = proto-oncogene serine/threonine-protein kinase; RAS = rat sarcoma viral oncogene; RTK = receptor tyrosine kinase; SHP2 = Src homology region 2 domain-containing phosphatase-2; SOS = son of sevenless. Parts of the figure were drawn using pictures from Servier Medical Art, licensed under CC BY 4.

The proliferation and angiogenesis of malignant astrocytomas in humans depends on signaling through the RAS network, and common changes observed in gliomas include EGFR amplification and activating mutations, early activation of receptor tyrosine kinase signaling, *NF-1* deletion, and increased levels of p21 signaling [58]. Among the several nuclear transcriptions factors that ERK targets in tumors with *RAS* mutations, MYC stands out. MYC’s critical function in modulating the activity of ERK in initiating and promoting cancer development in *RAS*-mutant gliomas has been demonstrated [59]. RAS is capable of activating cell signaling, promoting tumor progression through different signaling pathways such as RAF/MEK/ERK and PI3K/AKT [60]. A study by Zhao et al. demonstrated that the KRAS-ERK axis is responsible for positively regulating CD44 expression in response to radiation in GBM, as well as negatively regulating the expression of microRNAs miR-185 and miR-202. Expression of CD44 promotes *SRC* activation, a physiological proto-oncogene, a protein–tyrosine kinase that plays crucial roles in signaling pathways related to cell growth, division, migration and survival [61] leading to epithelial–mesenchymal transition in GBM cells [62]. Figure 1 summarizes oncogenic signaling pathways affected by mutant RAS and how they may relate to the development of CNS tumors.

## 4. Indirect Activation of RAS in CNS Tumors

In CNS tumors, RAS proteins are often aberrantly activated through indirect mechanisms, even in the absence of mutations in *RAS* genes themselves. One common route involves the loss of neurofibromin, a GTPase-activating protein encoded by the *NF-1* gene, which normally acts to inactivate RAS by accelerating GTP hydrolysis. Inactivating mutations in *NF-1*, found in a subset of gliomas, lead to persistent RAS signaling. Another frequent mechanism is the overactivation of receptor tyrosine kinases (RTKs) such as EGFR and PDGFR. These receptors, when overexpressed or mutated—as commonly observed in gliomas—enhance upstream signaling that indirectly stimulates RAS activity. Epigenetic silencing of genes encoding RAS inhibitors, such as RASAL, through promoter methylation also contributes to sustained RAS activation by disrupting its regulation. Additionally, dysregulation of microRNAs that target RAS signaling components can amplify pathway activity. These non-mutational forms of RAS activation highlight the complexity of signaling dysregulation in CNS tumors and offer alternative avenues for therapeutic intervention [35,63].

Loss of neurofibromin in low-grade gliomas (ex-Pilocytic astrocytoma) leads to activation of the RAS/RAF/MEK/ERK pathway, but there is rarely an aggressive progression, and this may be correlated with immunological or microenvironmental regulation [64]. High-grade gliomas, when *RAS* hyperactivation due to loss of neurofibromin can be combined with other mutations such as *TP53*, *ATRX*, *CDKN2A*, thus promote aggressive proliferation [65]. In medulloblastomas, according to the evidence, aberrant activation of RAS can influence the SHH (Sonic Hedgehog) pathway, promoting tumor growth [66].

Given its aggressiveness and molecular complexity, GBM has been the central focus of RAS pathway studies in CNS tumors, however it is important to recognize the broader spectrum of CNS tumor subtypes and their distinct mechanisms of RAS pathway dysregulation. For instance, medulloblastomas have been shown to activate RAS signaling via alterations in the SHH and WNT pathways, despite lacking canonical *RAS* mutations [66]. In oligodendrogliomas and anaplastic astrocytomas, rare RAS mutations (e.g., *KRAS^G12A^*, *NRAS^Q61R^*) have been reported, but functional dependency is often driven by co-occurring events such as 1p/19q codeletion or *IDH* mutations [13,41]. These subtype-specific patterns underscore the need to evaluate RAS pathway activation beyond mutational status, incorporating epigenetic regulation, feedback signaling, and tumor microenvironmental cues [35,63,66].

## 5. KRAS and Glioblastoma

GBM is the primary malignant brain tumor with the highest glioma prevalence in adults [67]. Based on the WHO’s classification of malignancy, it is a grade 4 tumor [1], which is known for its ability to rapidly infiltrate brain tissues, and being treated with ionizing radiation, which can frequently result in acquired resistance [68]. According to the WHO classification, diffuse astrocytic tumors are classified based on the *IDH* mutational status, where the presence or absence of this mutation plays a crucial role in the differentiation and prognosis of these gliomas. Diffuse gliomas that affect the adult age group are astrocytoma *IDH*-mutated; oligodendroglioma *IDH*-mutated and 1p/19-codeleted; and GBM *IDH*-wildtype [1]. Therefore, the presence or absence of an *IDH* mutation is decisive for the current diagnosis.

The most common genetic alterations in gliomas are *EGFR* (epidermal growth factor receptor) and *MDM2* (murine double minute 2) amplifications, *TP53*, *PTEN* (phosphatase and tensin homolog) and *IDH* 1/2 mutations, loss of heterozygosity (LOH) of chromosome 10q, 1p/19q codeletion and *MGMT* (O_6_-methylguanine-DNA methyltransferase) promoter methylation [33]. In the case of GBM, the presence of homozygous deletion of *CDKN2A/B*, mutation of the *TERT* promoter, amplification of the *EGFR* gene, alterations in the number of copies of chromosomes 7 and 10 (+7/−10), microvascular proliferation and necrosis are the characteristics that result in the highest classification grade [1,69]. The average annual incidence rate of primary GBM differs greatly between different age groups and is associated with older age, accounting for only 3% of CNS tumors in the 0–19 age group. The average age at diagnosis is around 64 years, and it has its highest incidence in the 75 to 84 age group (15.03 per 100,000 people) [70,71].

GBM may present mutations in genes involved in activated KRAS signaling, such as NF-1 (neurofibromin-1), indicating KRAS pathways as a potential target in this tumor [72]. However, reports indicate that *RAS* mutations are not associated with any specific histological glioma subtype [13]. Cerebellar GBM has been reported to bear *RAS* mutations [73]. A study has suggested *NRAS* mutations as an important molecular alteration that leads to aberrant RAS signaling in GBM [74]. The *KRAS^G^*^12D^ mutation alone does not initiate glioma formation, but seems to be required for progression to high-grade tumors such as GBM [39]. Overexpression of *KRAS* mutations have been demonstrated to modulate cisplatin resistance in human GBM cell culture [75].

Studies using animal models showed that GBM tumorigenesis is induced after *KRAS* activation [76]. In addition, RAS can be activated as a result of overexpression and/or activation mutations in other genes that act on RAS, such as *EGFR* [8]. In addition, it has also been shown that *KRAS* is important for maintaining GBM tumor development in vivo [77]. Approximately 10% of GBM tumors exhibit inactivating alterations in NF-1 leading to hyperactive RAS activity [78]. Collectively, these reports suggest a relationship between RAS pathways and GBM.

**Figure 2:**
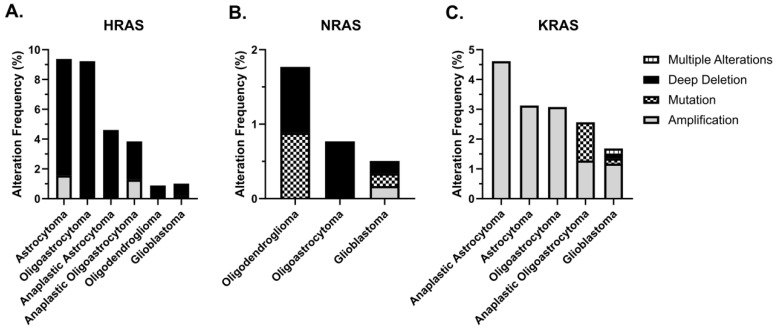
*RAS* alteration frequencies in gliomas. Using data retrieved from cBioPortal (accessed on 4 February 2025), the frequency of *RAS* alterations was analyzed across glioblastoma and lower-grade gliomas. The observed alterations, including amplification, deletion, mutation, and multiple alterations, were examined in various glioma subtypes; (**A**) frequency of *HRAS* alterations, (**B**) *NRAS* alterations, and (**C**) *KRAS* alterations.

Using data retrieved from cBioPortal, we investigated RAS alteration frequency in GBM and lower-grade gliomas, which were compiled in Figure 2. Observed alterations include amplification, deletion, mutation and multiple alterations across astrocytoma, anaplastic astrocytoma, oligodendroglioma, anaplastic oligoastrocytoma, oligoastrocytoma and GBM samples. Among the 1122 patients tested, driver mutations were detected in *KRAS* in three cases and in *NRAS* in two cases, with mutation frequencies of 0.3% and 0.2%, respectively. All mutations were of the missense type. Specifically, one patient diagnosed with GBM harbored a *KRAS*^G12D^ mutation with a low-level copy number gain, another GBM patient had a *KRAS*^G12D^ mutation with high-level copy number amplification, one patient diagnosed with anaplastic oligoastrocytoma exhibited a diploid *KRAS*^K5E^ mutation, another GBM patient had a diploid *NRAS*^Q16L^ mutation, and one patient diagnosed with oligodendroglioma showed an *NRAS*^Q16K^ mutation with shallow copy number deletion. Identifying RAS pathway activation in response to different *RAS* mutations can guide eligibility for targeted therapies, such as MEK or KRAS inhibitors. This approach may support personalized treatment planning, real-time monitoring, and improved prognostic assessment in gliomas management.

## 6. Strategies to Inhibit Mutated *RAS*

### 6.1. Disrupting Post-Translational Lipid Modifications at the RAS C-Terminal

RAS proteins feature a conserved CAAX sequence, where C represents cysteine, A is typically an aliphatic amino acid, and X denotes any amino acid. This sequence directs the post-translational modification of the protein’s C-terminal by attaching a polyisoprenoid lipid, typically a farnesyl group. This lipidation converts the otherwise hydrophilic RAS protein into a membrane-associated molecule, a crucial step for its activation and downstream signaling [79].

Initial efforts inhibited RAS-targeted post-translational lipid modifications at its C-terminal, a region critical for its maturation and membrane localization. Specifically, inhibitors of farnesyltransferase (FT), the enzyme responsible for lipid attachment, showed promise in preclinical trials for *HRAS*-mutant tumors. However, these inhibitors failed in clinical trials due to alternative prenylation of KRAS isoforms by geranylgeranyl transferase, a mechanism bypassing FT inhibition [80].

### 6.2. Inhibitors of the RAS–Effector Interaction

Major KRAS–effector pathways that are targeted by drugs comprise the MAP–kinase (MAPK) pathway, such as BRAF and MEK. Such mutations are common to various types of cancer, including melanoma, lung cancer, colorectal and thyroid carcinoma. Trametinib is a MEK inhibitor used in the treatment of melanomas with *BRAF*^V600E/K^ mutations [81], Cobimetinib is a MEK inhibitor used in combination with Vemurafenib for the treatment of advanced melanoma [82], Selumetinib is used in tumors with *NRAS* mutations and in radioiodine-refractory thyroid carcinomas [83], while Binimetinib has shown potential in melanomas and other *KRAS*/*NRAS*-mutant solid tumors [84]. Additionally, the Encorafenib + Binimetinib combination is used to treat *BRAF*-mutated melanomas [85].

Inhibiting SOS1, a KRAS activator and crucial feedback regulator, has emerged as a promising strategy for the treatment of KRAS-driven cancers. Hofmann et al. identified BI-346, a potent, selective and orally bioavailable SOS1 inhibitor. By binding to the catalytic domain of SOS1, BI-346 blocks its interaction with KRAS, reducing the formation of GTP-bound RAS and cellular proliferation in various types of KRAS-driven cancers. Notably, BI-346 reduces feedback reactivation caused by MEK inhibitors, thereby increasing the sensitivity of KRAS dependent cancers to MEK inhibition. This combination of SOS1 and MEK inhibition represents a novel and effective therapeutic concept for treating KRAS associated tumors [86].

Tropomyosin receptor kinases (TRK) are a subgroup of receptor tyrosine kinases (RTKs) crucial for brain development. In pediatric high-grade gliomas, TRK gene fusions frequently serve as the sole oncogenic drivers and may initiate tumorigenesis, activating both the PI3K-AKT and RAF-MEK-ERK pathways [87,88,89]. Clinical trials involving TRK-specific tyrosine kinase inhibitors (TKIs) have shown promising response rates in TRK fusion-positive primary and metastatic brain tumors [90]. However, these responses are often brief due to the emergence of resistance mechanisms, particularly mutations impacting downstream pathway effectors like RAS, underscoring their significant role in shaping treatment outcomes [91].

Several promising drugs targeting the RAS pathway are currently in preclinical and clinical development. These include NHTD inhibitors, such as ((E)-N′-((3-(tert-butyl) 2-hydroxy-6,7,8,9-tetrahydrodibenzo [b,d] furan-1-yl) methylene-2,4-dihydroxybenzohydrazide, which specifically target key proteins in the RAS signaling cascade. Additionally, compounds like Deltrazin, Deltazinones, and Deltasonamides aim at upstream proteins of the RAS cascade, with potential targets including PDEδ, SHP2, and SKT19. Direct strategies to target *RAS* mutations include covalent inhibition of the G12C mutation using inhibitors such as Kobe2602, Kobe0065, and Rigosertib. These approaches exploit unique structural features of *RAS* mutants, paving the way for selective and effective therapy [92].

MicroRNAs notoriously act as tumor suppressors by targeting key signaling pathways. For example, miR-124 is downregulated in oligodendroglioma, astroblastoma, GBM cell lines and in the plasma of glioma patients. Its loss promotes angiogenesis and invasiveness; therefore, it shows potential as a diagnostic and prognostic marker for brain tumors [93]. Interestingly, NRAS is a target of tumor suppressor miR-515-5p, which is downregulated in GBM biopsies, indicating the oncogenic role of NRAS in this tumor. Regarding therapy, it has been proposed that circular RNA SMARCA5-mediated upstream control of miRNAs could be applied as targeted molecular therapy [94].

### 6.3. Covalent Inhibition of KRAS^G12C^

*KRAS* has a hotspot mutation at codon G12, *KRAS*^G12C^, which is the third most common mutation at this position. In codon 12 of *KRAS*, there is a reactive cysteine that can be exploited for the development of small inhibitory molecules. Therefore, covalently targeting the cysteines of the active site is a broad strategy employed in the development of new drugs. Ostrem and colleagues studied potential RAS inhibitors by, initially, testing the covalent inhibition of *KRAS*^G12C^ to circumvent the toxicity associated with inhibiting RAS isoforms. In fact, *KRAS*-WT does not have cysteines in the active site, thus allowing specific inhibition of *KRAS^G12C^* through this covalent method [95].

Among the therapeutic agents targeting dysfunctional RAS is compound 3144; a small molecule is the most promising candidate inhibitor, which binds to the conserved ASP38 residue in the switch-I region, blocking RAS–effector binding. While it inhibits *KRAS*^G13D^ tumor growth in vivo, off-target activity and associated toxicity remain challenges [96]. Covalent inhibitors such as Sotorasib irreversibly bind to the cysteine at position 12 of mutated *KRAS*, blocking its oncogenic activity [97]. Similarly, Adagrasib has demonstrated promising clinical trial results, further validating covalent inhibition as a viable strategy against *KRAS^G12C^* mutations [98].

### 6.4. Current Clinical Trials Involving CNS Tumors and RAS

While direct *RAS* mutations are uncommon in CNS tumors, targeting the RAS pathway remains a potential therapeutic strategy. For example, oncolytic viruses like pelareorep have been explored for their ability to selectively infect and kill tumor cells with activated RAS pathways [99]. Clinical trials for RAS-related alterations in the CNS are very scarce, with currently only two clinical trials ongoing. One of them is titled “Expanded Access to Ulixertinib (BVD-523) in Patients with Advanced MAPK Pathway-Altered Malignancies”. The study aims to provide Ulixertinib (BVD-523) for compassionate use in patients with advanced cancer whose solid tumors have been affected by the MAPK pathway, such as *KRAS*, *NRAS*, *HRAS*, *BRAF*, *MEK*, and *ERK* mutations, which do not fully respond to current treatment or have exhausted their available therapeutic options. With Ulixertinib being able to be used as monotherapy or in combination with other clinically acceptable agents, conditionally approved by the drug manufacturer (ID NCT04566393) (ClinicalTrials.gov ID, NCT04566393).

Another ongoing clinical trial is entitled “Safety and Efficacy of NEO212 in Patients with Astrocytoma *IDH*-mutant, GBM *IDH*-wildtype or Brain Metastasis”. It is a phase 1/2 multicenter clinical study that aims to investigate the safety, pharmacokinetics, and efficacy of the repeated dose regimen of NEO212 in treating patients with advanced-stage mutant *IDH* astrocytoma, *IDH*-wildtype GBM and uncontrolled brain metastases confirmed by imaging exams [100].

The recently completed TADPOLE trial (NCT02684058) indicates that targeted therapy focusing on the RAS/RAF/MAPK signaling pathway is beneficial for pediatric brain tumors with *BRAF* mutations. Dabrafenib in combination with Trametinib has shown significant efficacy and tolerability in treating pediatric low-grade gliomas (LGG) with *BRAF*^V600E^ mutations (NCT01677741). The FDA approved Dabrafenib and Trametinib for pediatric LGG in children aged one or older in 2023, and for relapsed/refractory BRAF^V600^-mutant solid tumors in patients aged six and older in 2022, underscoring its clinical effectiveness [78]. Considering that most drugs are developed for metastatic tumors, a summary of current clinical trials targeting RAS pathways in mostly brain metastases and some gliomas is shown in Table 1.

## 7. Materials and Methods

The present review provides an overview of the existing literature for detected *RAS* mutations in CNS tumors, exploring their potential role as key oncogenes and detailing current clinical trials. To achieve this, we conducted a comprehensive search of the MEDLINE database via PubMed for articles published in English up until February of 2025. Key Medical Subject Headings (MeSH) and free-text search terms, including “RAS”, “Central nervous system tumors”, “Brain cancer”, “KRAS”, “NRAS”, “HRAS” and “RAS mutations” were used to identify relevant studies. Both original research and review articles were selected for analysis, focusing on data related to the detection of RAS mutations in CNS tumors. To ensure a comprehensive examination, additional publications were retrieved from the references of selected articles.

Data on alterations in *KRAS*, *NRAS* and *HRAS* in gliomas were queried via cBioPortal (https://www.cbioportal.org/, accessed on 4 February 2025) [111,112], using The Cancer Genome Atlas Program’s (TCGA) Firehose Legacy study for the datasets named “Glioblastoma Multiforme” and “Brain Lower Grade Glioma”, containing 604 and 530 samples, respectively. In the Cancer Types Summary and Mutations tabs, lists containing Cancer Type Detailed, Protein Change, Mutation Type, Alteration Frequency and Copy Number Variation information were downloaded for both cohorts. Graphs were generated with the data taken from cBioPortal using GraphPad Prism software version 9.0.0 (GraphPad Prism Software, Inc., La Jolla, CA, USA).

Furthermore, clinical trials were reviewed using the ClinicalTrials.gov database. Search terms such as “Brain metastasis”, “Central Nervous System tumors”, “Glioma”, “Glioblastoma”, “RAS mutations”, “KRAS”, “HRAS”, “NRAS” and “RAS signaling pathways” were used to locate ongoing trials investigating RAS-related trials involving brain metastasis and glioma treatment. The findings from these trials were incorporated into a dedicated section of this review to highlight recent clinical research targeting *RAS* and *RAS*-related mutation end pathways in CNS tumor management.

## 8. Conclusions

While progress has been made in identifying therapeutic targets for RAS-affected tumors, significant challenges remain. Although *RAS* mutations are present in some CNS tumors, they are not considered key biomarkers due to their low or sporadic expression. However, the role of RAS in the CNS remains underexplored, creating a major knowledge gap in understanding its oncogenic potential. Notably, KRAS is expressed in GBM cells, with studies reporting varying expression levels. While direct *RAS* mutations are not very frequent, RAS-related signaling pathways are frequently activated in certain CNS tumors such as GBM, through alternative mechanisms like EGFR amplification, NF1 loss, and PDGFR overexpression, which converge on the RAS/RAF/MEK/ERK and PI3K/AKT axes. These indirect modes of RAS activation suggest that RAS itself may not be the primary oncogenic driver, yet its downstream pathways contribute significantly to tumor progression and therapeutic resistance. Therefore, further research is essential to elucidate the role of RAS itself, along with its associated pathway alterations, in the initiation and progression of CNS tumors, as well as to assess their potential as therapeutic targets in these tumors.

## Figures and Tables

**Table 1:** Current clinical trials involving RAS pathways in brain metastasis and glioma.

Trial Name	Therapy Used	ID	Reference
A Phase II Study evaluating intracranial efficacy of JDQ443 in Patients With KRAS G12C+ NSCLC and Brain Metastases	Opnurasib (JDQ-443)	NCT05999357	[101]
A Phase I/II Study of AMG 510 in Combination with MVASI in Patients with Advanced, Unresectable or Metastatic KRAS G12C Mutant NSCLC With Asymptomatic Brain Metastasis.	Sotorasib (AMG510) MVASI	NCT05180422	[102]
Phase II Study of Adagrasib + Stereotactic Radiosurgery (SRS) for Patients with Metastatic KRAS G12C-mutated NSCLC With Untreated Brain Metastases	Adagrasib Stereotactic Radiosurgery (SRS)	NCT06248606	[103]
A Phase 1, Open-label Study of Oral BDTX-4933 in Patients With KRAS, BRAF and Other Select RAS/MAPK Mutation Positive Neoplasms	BDTX-4933	NCT05786924	[104]
Study of Regorafenib in Combination with Oral Methotrexate for KRAS Mutated Non-Small Cell Lung Cancer (NSCLC)	Methotrexate Regorafenib	NCT03520842	[105]
Genomically-Guided Treatment Trial in Brain Metastases	Abemaciclib Paxalisib Entrectinib Adagrasib (MRTX849)	NCT03994796	[106]
Expanded Access to Ulixertinib (BVD-523) in Patients with Advanced MAPK Pathway-Altered Malignancies	Ulixertinib (BVD-523)	NCT04566393	[107]
Safety and Efficacy of NEO212 in Patients with Astrocytoma IDH-mutant, Glioblastoma IDH-wildtype or Brain Metastasis	NEO212 Ipilimumab Pembrolizumab Nivolumab Regorafenib Carboplatin Paclitaxel FOLFIRI Protocol Bevacizumab	NCT06047379	[108]
Study of Efficacy and Safety of Dabrafenib in Combination with Trametinib in Pediatric Patients with BRAF V600 Mutation Positive LGG or Relapsed or Refractory HGG Tumors	Dabrafenib Trametinib Carboplatin Vincristine	NCT02684058	[109]
A Study to Determine Safety, Tolerability and Pharmacokinetics of Oral Dabrafenib in Children and Adolescent Subjects	Dabrafenib	NCT01677741	[110]
